# Why Asplenic Patients Should Not Take Care of the Neighbour's Dog? A Fatal Course of *Capnocytophaga canimorsus* Sepsis

**DOI:** 10.1155/2018/3870640

**Published:** 2018-08-02

**Authors:** Patrick Langguth, Lothar Leissner, Günther Zick, Arno Fischer, Christiane Stuhlmann-Laiesz, Mona Salehi Ravesh, Friederike Austein, Olav Jansen, Marcus Both

**Affiliations:** ^1^Department of Radiology and Neuroradiology, University Hospital Schleswig-Holstein, Kiel, Germany; ^2^Department of General and Thoracic Surgery, University Hospital Schleswig-Holstein, Kiel, Germany; ^3^Department of Anesthesiology and Surgical Intensive Care, University Hospital Schleswig-Holstein, Kiel, Germany; ^4^Institute for Infection Medicine, University Hospital Schleswig-Holstein, Kiel, Germany; ^5^Institute for Pathology, University Hospital Schleswig-Holstein, Kiel, Germany

## Abstract

*Capnocytophaga canimorsus* (CC) belongs to the family Flavobacteriaceae which physiologically occurs in the natural flora of the oral mucosa of dogs and cats. In patients with a compromised immune system, CC can induce a systemic infection with a fulminant course of disease. Infections with CC are rare, and the diagnosis is often complicated and prolonged. We describe a patient with a medical history of prior splenectomy who presented with an acute sepsis and disseminated intravascular coagulation (DIC) and was initially treated on Waterhouse–Friderichsen syndrome (WFS). After the patient had died despite forced treatment in the intermediate care unit, the differential diagnosis of CC was confirmed by culture of blood smears. Later on, a retrospective third-party anamnesis revealed that the patient had contact to his neighbour's dog a few days before disease onset. In conclusion, patients with CC infection can mimic WFS and therefore must be included in the differential diagnosis, especially in patients with a corresponding medical history of dog or cat bites, scratches, licks, or simple exposure.

## 1. Case History

A 49-year-old man was brought from his home environment to hospital by the emergency medical service with acute epigastric pain accompanied by vomiting and diarrhoea. The symptoms had started quite suddenly a few hours previously and had persisted.

**Figure 1:**
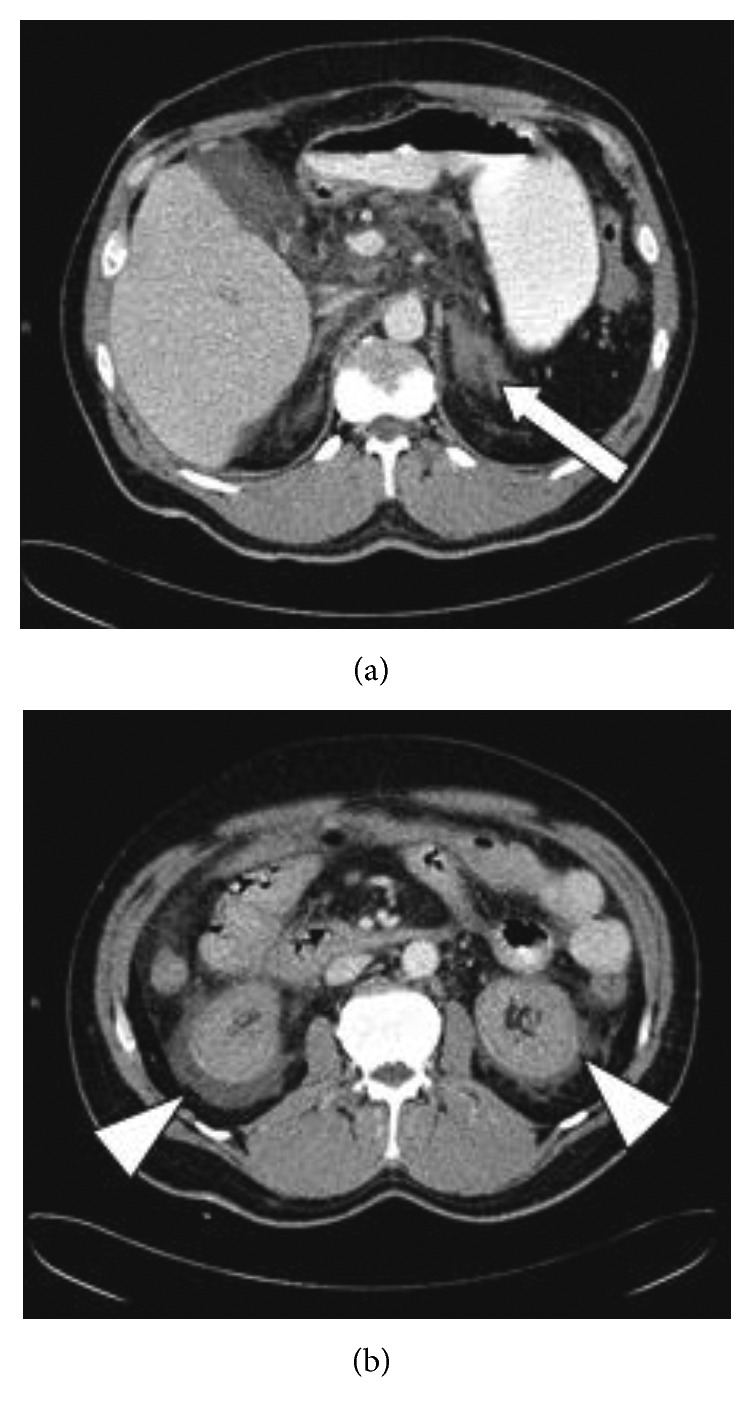
Contrast-enhanced abdominal CT demonstrates haemorrhage of the swollen adrenal glands (arrow) and status after splenectomy (a). There is fluid collection in the perirenal space (arrowheads), and the corticomedullary differentiation of the kidneys is lost (b).

**Figure 2:**
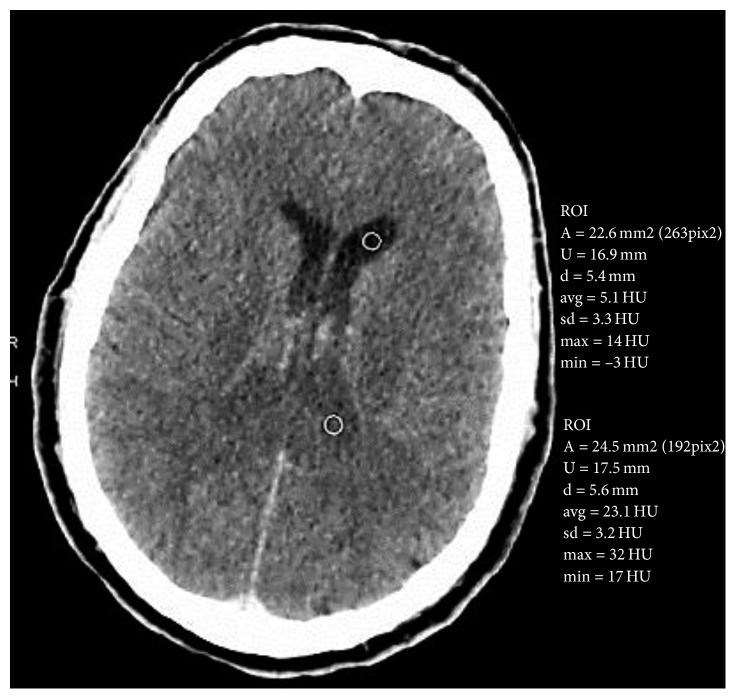
Cranial CT reveals hyperdense sedimentations (Hounsfield unit (HU) 23) in both posterior lateral ventricles, suggesting haemorrhage or pus.

On physical examination, a significantly pressure-sensitive stomach and multiple flea bites on both lower legs were found. The initial blood pressure of the patient was regular at 115/70 mmHg, and his pulse rate was 109/min. The patient had a normal body temperature of 36.7°C. His medical history documented a splenectomy after a motorcycle accident 30 years ago. A few hours after hospital admission, the patient felt dizzy, became febrile (38.9°C), and developed generalized oedema of the skin with petechiae and bleeding of the oral mucosa. Blood pressure decreased rapidly, so the patient was admitted to the intensive care unit (ICU) where he was intubated and monitored. The laboratory findings showed significant abnormalities: thrombocytopenia of 37 thrombocytes/nl, elevated D-dimer of 35 mg/l FEU, and severely impaired coagulation (INR 2.80, PTT > 120 sec). A high C-reactive protein of 82.9 mg/l was consistent with a severe infection, and the procalcitonin of 65.64 ng/ml was a clue to the fact that the problem had a bacterial source. The clinical and serological findings and the medical history of splenectomy justified the suspicion of sepsis of unknown origin, associated with an overwhelming postsplenectomy infection (OPSI) syndrome and disseminated intravascular coagulation (DIC). As the patient's condition was unstable, an empiric antibiotic therapy with cefotaxime and metronidazole was started after blood cultures were taken. DIC was treated with fresh frozen plasma (FFP), heparin, and prothrombin complex concentrate (PCC). Kidney failure had developed and required renal replacement therapy with continuous veno-venous haemodialysis (CVVHD). Owing to the continuously impaired clinical and laboratory parameters, thoracic and abdominal computed tomography (CT) was performed, which revealed diffuse ascites and oedema, especially in the perirenal space. The corticomedullary differentiation of the kidney was absent (Figure 1(b)). The adrenal glands were swollen on both sides, suggesting haemorrhage (Figure 1(a)). No abscess or other infectious foci were found. In a subsequent CT scan of the head, a hyperdense sediment in both lateral ventricles was observed with a Hounsfield unit (HU) of 23, also suggesting intraventricular pus or haemorrhage (Figure 2). In the preliminary synopsis, meningococcal sepsis was suspected with Waterhouse–Friderichsen syndrome (WFS). Lumbar puncture could not be performed to detect bacteria due to insufficient coagulation parameters.

As a result of examining the blood smears, which showed Gram-negative rods, the initial hypothesis of WFS could not be verified. Due to the microbiological findings, in particular the Gram-negative rods in the blood smear, a new suspicion of an infection with *Capnocytophaga canimorsus* (CC) arose, a bacterium that occurs physiologically in the oral mucosa of cats and dogs and can result in septic shock in immunosuppressed patients. It is difficult to microbiologically differentiate CC because this organism grows slowly in culture [1, 2]. Thus, our suspicion of an infection with CC could only be confirmed after two weeks by blood culture. In addition, an antibiotic resistance spectrum was determined.

**Figure 3:**
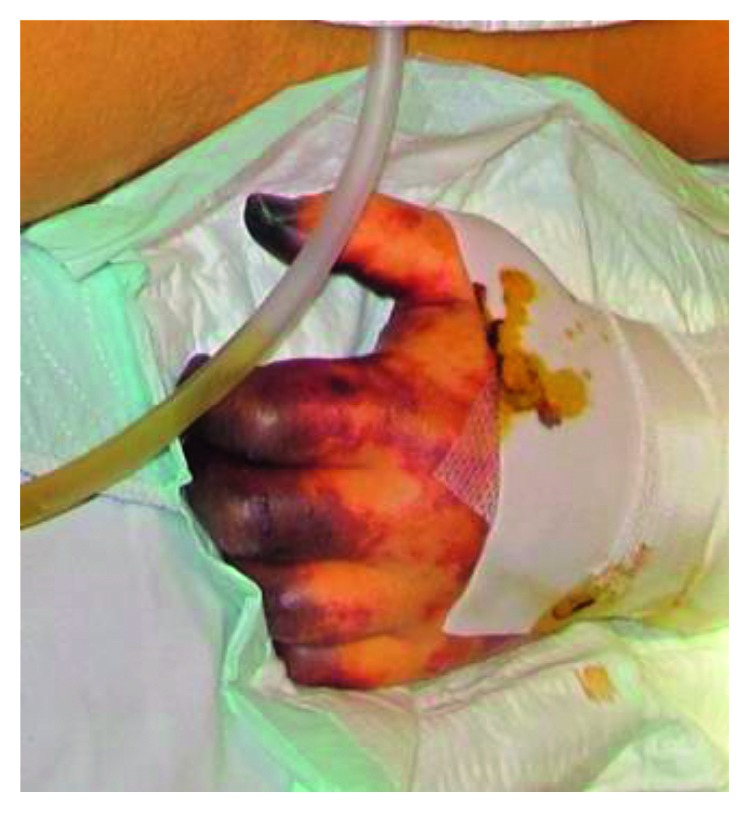
Necrosis of the left hand.

**Figure 4:**
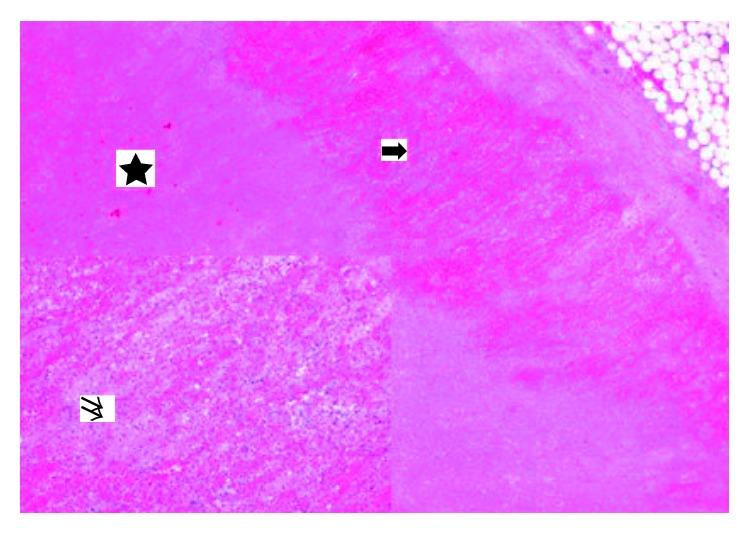
Haemorrhagic necrosis of the adrenal gland and complete necrosis of the adrenal gland. The different zones of the adrenal cortex cannot be clearly distinguished. Especially the cortex shows extensive haemorrhage (arrow). The cells are ill-defined and intermingled with fibrin exudation, showing nuclear shadows (insert, double arrow). The cellular compartment of the adrenal medulla is necrotic with intermingled fibrin exudation (star) (haematoxylin and eosin, original magnification 4x insert, original magnification 20x).

Despite forced treatment in the ICU, including vancomycin and meropenem, the patient's condition continued to deteriorate, accompanied by liver failure and severe necrosis in both legs and the left arm (Figure 3). Chest X-ray revealed pulmonary infiltrations. The patient died 15 days after hospitalization as a result of multiple organ failure. Consistent with the clinical presentation of a multiorgan failure, the autopsy showed signs of disseminated coagulopathy with extensive haemorrhagic necrosis of several organs. In particular, haemorrhagic necrosis of the lungs, the kidney and adrenal gland on both sides, the liver, the pancreas, and the small intestine and segmental necrosis of the large intestine were detected. These findings were confirmed by the histomorphological examination of tissue samples. Compatible with the morphological presentation of the adrenal gland in the CT scan and the macroscopic aspect, the histomorphological findings revealed complete haemorrhagic necrosis of the parenchyma on both sides (Figure 4).

**Figure 5:**
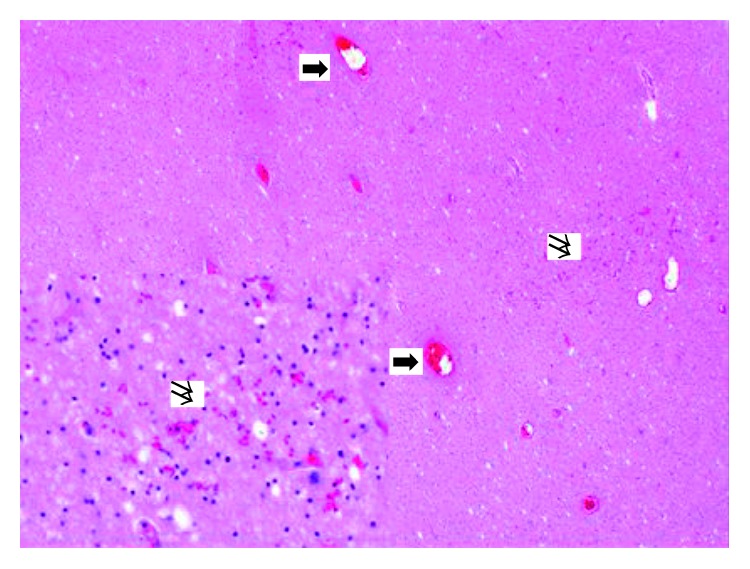
Dilated vessels, disseminated haemorrhage, and oedema of the brain. The brain shows ubiquitously oedema with loosened parenchyma. The vessels are dilated (arrows). Diffuse erythrocyte extravasation is shown (double arrows; haematoxylin and eosin, original magnification 4x insert, original magnification 20x).

The macroscopic and microscopic neuropathological examination of the brain and tissue samples showed dilated vessels, ubiquitously disseminated haemorrhage, and oedema (Figure 5). The clinically suspected meningitis could not be confirmed.

**Figure 6:**
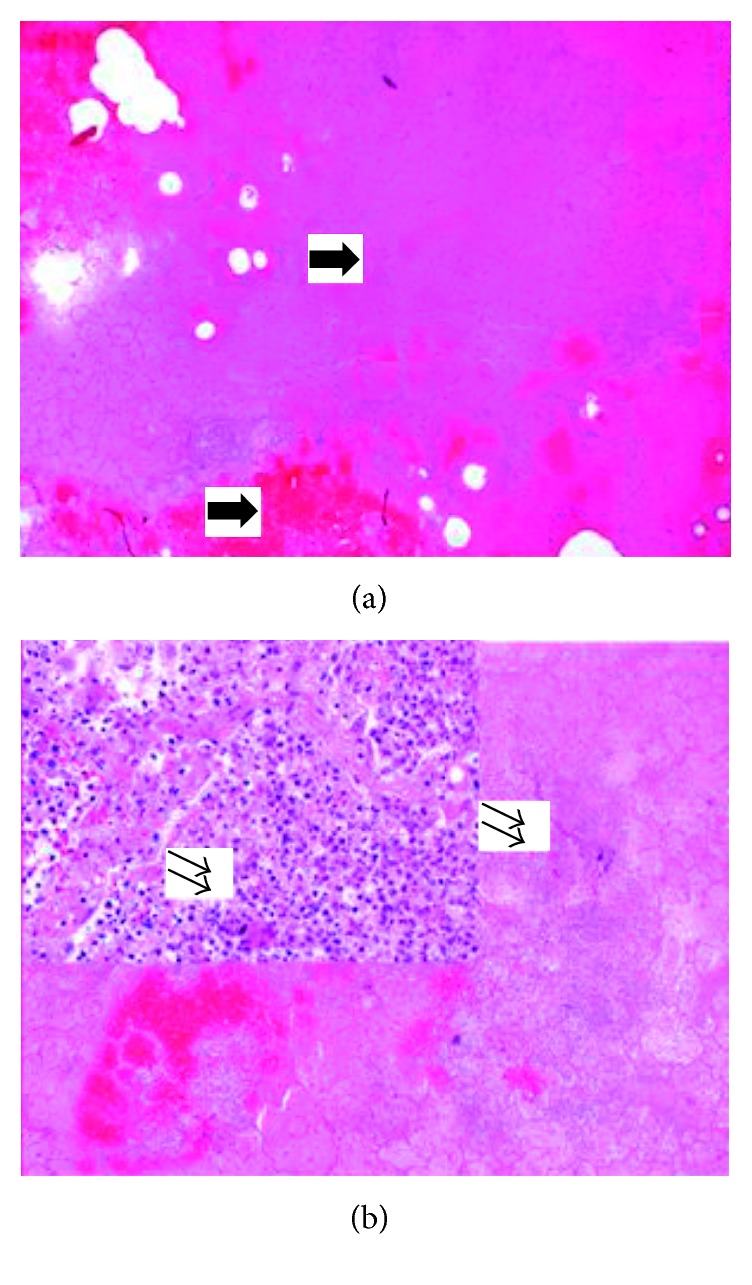
Complete necrosis of the lung with extensive haemorrhage and absceding bronchopneumonia. (a) Completely necrotic lung parenchyma with extensive intra-alveolar haemorrhage. The lung parenchyma is visible as a shadow. (b) An intra-alveolar infiltration by neutrophilic granulocytes (double arrows), focally with abscess formation (haematoxylin and eosin, original magnification 4x insert, original magnification 20x).

According to the performed ventilation, the lungs had signs of an acute respiratory distress syndrome (ARDS), and consistent with the pulmonary infiltrations visible on the chest X-ray, an ascending bronchopneumonia with accompanying fibrinous pleuritis was observed (Figure 6). In the synopsis of all results, the patient ultimately died as a result of septic multiorgan failure. Although the source of infection was still unknown, a retrospective third-party anamnesis revealed that the patient had taken care of his neighbour's dog a few days before the onset of the disease. Thus, with the many flea bites on both lower legs on initial physical examination, the dog can therefore be assumed as the carrier of the bacteria and also the cause of the patient's disease.

## 2. Discussion

*Capnocytophaga canimorsus* was first described in 1976 by Bobo and Newton [1] and is a slow-growing, Gram-negative, and rod-shaped bacterium belonging to the natural flora of the oral mucosa of dogs and cats. *Capnocytophaga* is exclusively found in the oral cavities of mammals [3]. This genus belongs to the family Flavobacteriaceae in the group Bacteroidetes [3, 4]. *C. canimorsus* has low virulence in healthy people, whereas a fulminant course of disease in people with a poor immune system, especially after splenectomy or chronic alcohol abuse, has been described [5–7]. Lion et al. reported that 33% of septicaemia occurs in asplenic patients [5]. Gaastra and Lipman described as possible transmission routes direct contamination by dog or cat bites, scratches, licks, and also the simple exposure to dogs and cats [8, 9]. It is known that up to 56% of affected patients have been bitten by an animal [2, 5, 6, 10]. The infection becomes manifest commonly 2 to 3 days after exposure, but symptoms can appear later, after up to 2 to 4 weeks [6].  Table 1 demonstrates the variety and usually nonspecific range of symptoms, including the general clinical symptoms of a systemic infection as well as the signs of sepsis with DIC, manifestations of the skin such as petechiae or purpura, meningitis, endocarditis, and multiple organ failure [5, 11]. As in our patient, the initial symptoms were abdominal pain, nausea, and vomiting. Since first isolated in 1976, the rate of infection is still low, but it is being seen with increasing frequency [2]. Pers et al. published an incidence of CC infections in Denmark that was estimated to be 0.5 cases per 1 million people per year and described in the study an average age of 59 years with a range from 28 to 83 [7]. That study confirms the results of other authors, showing that patients in middle or old age are most commonly involved, as in the case reported here [5–7]. In fact, patients who are suspected of having an infection with *C. canimorsus* should be specifically asked about exposure to dogs and cats, bite and scratch injuries should be searched for, and immune deficiencies must be elicited. In addition, a microbiological examination of blood, secretions from the throat, and cerebrospinal fluid should be performed. Imaging findings in patients with CC are not well reported in the literature. In the present case, the CT scans demonstrated, in the posterior horns of both lateral ventricles, signs of inflammation or bleeding as well as an adrenal haemorrhage, which is a known diagnostic constellation for WFS. Indeed, the clinical and radiological presentation of a CC infection is very similar to that of WFS [12]; thus, both infections should be included in the differential diagnosis. WFS was first described in 1911 by Rupert Waterhouse and is characterized by petechial rash, coagulopathy, cardiovascular collapse, and bilateral adrenal haemorrhage [13]. In 90% of cases, meningococcal bacteria (*Neisseria meningitidis*) are detected, but other pathogens, such as *Neisseria gonorrhoeae*, *Escherichia coli*, *Haemophilus influenzae* type B, and *Staphylococcus aureus*, are described in the literature [14] and must also be considered in the diagnosis. Mortality is high for both infections, but especially for *C. canimorsus* (33%), whereby the prognosis of concomitant septic shock or DIC is vastly worse [5]. Considering that all symptoms at disease onset tend to be nonspecific, the radiological examination, especially CT, is an important tool for early diagnosis and initiation of therapy to prevent fulminant disease courses such as sepsis and DIC by early intervention.

## 3. Conclusions

In summary, CC infection can present clinically and in imaging findings as WFS and therefore must be included in the differential diagnosis, especially in patients with a corresponding medical history of dog or cat bites, scratches, licks, flea bites, or simple exposure. Although previously healthy people can suffer from an infection with *Capnocytophaga canimorsus*, too, immunocompromised and asplenic patients in particular are at higher risk.

## Figures and Tables

**Table 1:** The most common symptoms of an infection with *C. canimorsus*.

Symptoms	Frequency (%)
Fever	37–92
Abdominal pain/diarrhoea	21
Nausea/vomiting	18
Headache	18
Muscle pain	<10
Petechiae	35
DIC with fulminant sepsis	13–38
Necrosis/gangrene	10
Endocarditis	7–15
